# Monocytic Ontogeny of Regenerated Macrophages Characterizes the Mesotheliomagenic Responses to Carbon Nanotubes

**DOI:** 10.3389/fimmu.2021.666107

**Published:** 2021-06-14

**Authors:** Micaela Orsi, Mihaly Palmai-Pallag, Yousof Yakoub, Saloua Ibouraadaten, Michèle De Beukelaer, Caroline Bouzin, Bertrand Bearzatto, Jérôme Ambroise, Jean-Luc Gala, Davide Brusa, Dominique Lison, François Huaux

**Affiliations:** ^1^ Louvain Centre for Toxicology and Applied Pharmacology (LTAP), Institute of Experimental and Clinical Research (IREC), Université Catholique de Louvain (UCLouvain), Brussels, Belgium; ^2^ Imaging Platform, Institute of Experimental and Clinical Research (IREC), Université Catholique de Louvain (UCLouvain), Brussels, Belgium; ^3^ Center for Applied Molecular Technologies, Institute of Experimental and Clinical Research (IREC), Cliniques Universitaires Saint-Luc and Université Catholique de Louvain (UCLouvain), Brussels, Belgium; ^4^ Flow Cytometry Platform, Institute of Experimental and Clinical Research (IREC), Université Catholique de Louvain (UCLouvain), Brussels, Belgium

**Keywords:** macrophage origin, macrophage niche, macrophage proliferation, peritoneal macrophages, mesothelioma, inflammation, monocytes

## Abstract

Macrophages are not only derived from circulating blood monocytes or embryonic precursors but also expand by proliferation. The origin determines macrophage fate and functions in steady state and pathological conditions. Macrophages predominantly infiltrate fibre-induced mesothelioma tumors and contribute to cancer development. Here, we revealed their ontogeny by comparing the response to needle-like mesotheliomagenic carbon nanotubes (CNT-7) with tangled-like non-mesotheliomagenic CNT-T. In a rat peritoneal cavity model of mesothelioma, both CNT induced a rapid macrophage disappearance reaction (MDR) of MHCII^low^ resident macrophages generating an empty niche available for macrophage repopulation. Macrophage depletion after mesotheliomagenic CNT-7 was followed by a substantial inflammatory reaction, and macrophage replenishment completed after 7 days. Thirty days after non-mesotheliomagenic CNT-T, macrophage repopulation was still incomplete and accompanied by a limited inflammatory reaction. Cell depletion experiments, flow cytometry and RNA-seq analysis demonstrated that, after mesotheliomagenic CNT-7 exposure, resident macrophages were mainly replaced by an influx of monocytes, which differentiated locally into MHCII^high^ inflammatory macrophages. In contrast, the low inflammatory response induced by CNT-T was associated by the accumulation of self-renewing MHCII^low^ macrophages that initially derive from monocytes. In conclusion, the mesotheliomagenic response to CNT specifically relies on macrophage niche recolonization by monocyte-derived inflammatory macrophages. In contrast, the apparent homeostasis after non-mesotheliomagenic CNT treatment involves a macrophage regeneration by proliferation. Macrophage depletion and repopulation are thus decisive events characterizing the carcinogenic activity of particles and fibres.

## Introduction

Macrophages are the main immune cells recruited to clean up the body from foreign materials. They also contribute to the adverse effects of inhaled particles, and their fundamental role in particle-induced diseases like mesothelioma, is widely recognized (1). Mesothelioma is a cancer of mesothelial cells of peritoneum, pleura and pericardium with poor prognosis and limited therapies. Mesothelioma is mainly induced by chronic inhalation of asbestos fibres in human and by injection of carbon nanotubes (CNT) in rodent (2). As with asbestos fibres, the shape and the geometry of CNT determine their pathogenicity (3). Several studies demonstrated that needle-like shaped CNT (CNT-7) but not tangled CNT (CNT-T) rapidly induce peritoneal mesothelioma in rats (4). CNT-7 peritoneal injection is now accepted as the most simple, reproducible, and less time-consuming model to study mesothelioma (5).

Defining how mesothelioma-inducing particles shape macrophage fate and phenotype is a crucial challenge to develop new efficient therapies for mesothelioma and to investigate the carcinogenicity of particles and fibres. In the current study, we focus on the macrophage responses to mesotheliomagenic needle-like CNT (CNT-7) and non-mesotheliomagenic tangled CNT (CNT-T) in a rat peritoneal model (6). This original comparative model is not available by using natural asbestos fibres where all forms are carcinogenic.

As for asbestos, the first macrophage response to mesotheliomagenic CNT is recognition *via* the mannose (MR), toll-like (TLR) and scavenger receptors (SR), followed by internalization *via* phagocytosis (7). Downstream events comprise lysosome membrane permeabilization, reactive oxygen species secretion and activation of the NF-KB signalling pathway, pivotal for the release of neutrophil- (TNF-α and IL-6) and monocyte- (CCL2) chemotactic cytokines (8). Moreover, particle endocytosis results in the specific release by macrophages of active IL-1β *via* the NLRP3-inflammasome machinery (9). The inflammatory neutrophils recruited in CNT-induced damaged and inflammatory tissues represent an important driver of carcinogenesis by triggering genomic instability in mesothelial cells (10).

Chronic exposure combined with impaired clearance of CNT cause their accumulation in the lung and mesothelial cavities, where accumulated macrophages switch from a pro-inflammatory M1 to a pro-tumoral M2 phenotype (1). The skewing into M2 phenotype causes the dampening of T cell activity against cancer cells by producing immunosuppressive mediators (Arginase 1, TGF-β and IL-10), and the development of malignant responses (11). The intensity of macrophage responses to CNT is related to their physico-chemical properties: rigid and needle-like CNT impair macrophage clearance and trigger a pronounced and persistent release of inflammatory and immunosuppressive mediators (1, 8).

The exact origin and expansion pathways of macrophage populations after CNT exposure and during mesothelioma development is, however, almost unknown. While it has long been accepted that macrophages originate from bone marrow-derived circulating blood monocytes, new studies have extended this paradigm. Tissue macrophage subsets can also derive from yolk sac-related embryonic progenitors and be self-renewing cells, independent of hematopoietic progenitors (12).

Animal studies showed that the peritoneal cavity, in steady state conditions, houses two distinct macrophage subsets (13). The majority of resident macrophages (90 %) are large peritoneal macrophages (LPM), self-renewing cells arising from embryo-derived precursors and characterized, in the mouse, by the expression of F4/80, CD11b and CD163 but not MHCII. These cells maintain the homeostasis of the cavity (14). Upon homeostatic imbalance following an inflammatory stimulus, MHCII^low^ LPM macrophages disappear from the peritoneum (macrophage disappearance reaction or MDR), leaving empty spaces in the pattern. The repopulation of this empty niche is triggered by the sudden lack of proximity between the cells and by a higher disposability of growth factors for monocyte engraftment or macrophage proliferation (15). During the macrophage disappearance reaction, MHCII^low^ LPM macrophages can easily migrate from the peritoneal cavity to the near omentum, a visceral adipose tissue where they restore an already existing pool of proliferating macrophage progenitors (16). In contrast, small peritoneal macrophages (SPM) represent 10 % of the population in steady state conditions and are MHCII^high^ short-lived cells continuously replaced by CCR2^pos^ monocytes (13). During an inflammatory reaction, these cells are recruited from circulating monocytes and become the predominant population of the peritoneal cavity. SPM have higher phagocytic capacity than LPM for particles or bacteria (13).

The aim of this study is to determine the exact origin and the phenotype of macrophage populations in response to mesotheliomagenic particles. We used a relevant rat peritoneal model of mesothelioma to define the phenotype and origin (MHCII^high^ SPM *versus* MHCII^low^ LPM signature) of macrophage populations in response to mesotheliomagenic CNT-7 and non-mesotheliomagenic CNT-T (4).

Our results reveal, for the first time, a specific macrophage ontogeny during the early events of mesothelioma development. We show that monocyte-derived MHCII^high^ SPM-like macrophages dominate the early peritoneal responses to mesotheliomagenic particles.

## Materials and Methods

### Carbon Nanotubes

CNT-7 are multi-walled carbon nanotubes MWCNT-XNRI-7 from Mitsui & co (Ltd., Tokyo, Japan, Lot # 05072001 K28) sub-sampled at Norwegian Research Centre for the Working Environment (NRCWE) with the code NRCWE-006. CNT-T (tangled, kindly provided by Prof S. Toyokuni, Nagoya University, Japan) and their physico-chemical characteristics have been documented in detail previously (17). All particles were first treated at 200°C for 2 h to remove any possible trace of endotoxin and suspensions were prepared immediately before administration by sonication and manual vortexing in sterile phosphate-buffered saline (PBS) containing 1.4 mg bovine serum albumin/ml.

### Animals

Specific pathogen free (SPF) female Wistar rats (8 weeks old, 200 g) (RGD ID: 13792727) were purchased from Janvier SAS (St.-Berthevin, France). The animals were housed at the local animal facility under SPF conditions, with a 12-h light–dark cycle and controlled temperature and humidity. All animal experiments were approved by the local committee for animal research at the Université catholique de Louvain, Comité d’Ethique pour l’Expérimentation Animale, Secteur des Sciences de la Santé, Brussels, Belgium (No LA1230312). International recommendations for doses administered controls and group size were followed (18). The animals were injected intraperitoneally with the different particles suspended in a volume of 1 ml of PBS-BSA. They were given tap water and sterilized pellets ad libitum. Groups of 4 to 5 Wistar rats were injected with 2 mg of test material, corresponding to 0.67*109 WHO fibres of CNT-7 able to induce mesothelioma in rat {Cullen, 2002 #781;Huaux, 2016 #464}. Rats were then sacrificed after 3 hours and 1, 3, 7, 15 or 30 days. After the sacrifice, a volume of 25 mL of NaCl solution was intraperitoneally injected in rat to wash the cavity and sample fluid and leukocytes.

### Cell Analysis and Purification

**Figure 1:**
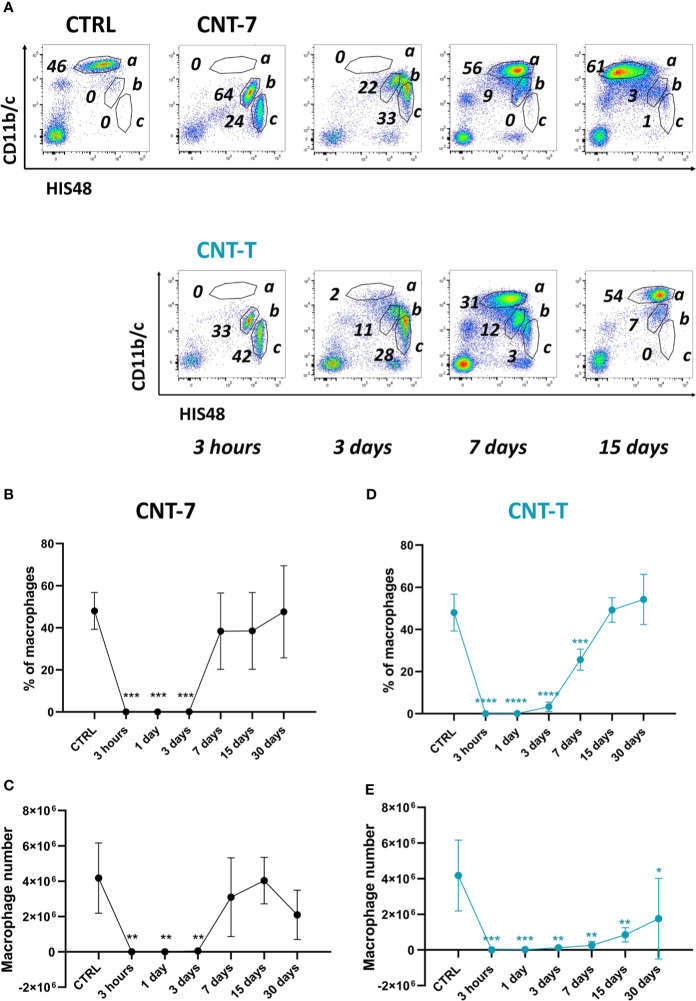
Disappearance reaction and repopulation of peritoneal macrophages after exposure to mesotheliomagenic CNT-7 and non-mesotheliomagenic CNT-T. **(A)** Flow cytometry dot plots (CD11b/c, His48) of peritoneal cells (a = macrophages, b = neutrophils, c = monocytes) of CTRL (untreated rats sacrificed at 3 hours) or CNT-treated Wistar rats (2 mg, I.P.) (3 hours, 3, 7, 15 and 30 days). Proportion **(B, D)** and total number **(C, E)** of peritoneal macrophages in response to CNT-7 and CNT-T (day 0-30). Each point represents the mean of 4 samples ± SD. The results were statistically analysed using one-way ANOVA test followed by Dunnett’s test. (*) p = 0.0332, (**) p = 0.0021, (***) p = 0.0002, (****) p < 0.0001 indicates a statistically significant difference with controls.

Total peritoneal cells were counted using a Burker cell chamber and peritoneal cell suspensions were stained using antibodies specific for CD11b/c-APC (Cat. # 201809, RRID: AB_313995) obtained from BioLegend or CD11b/c-BV421 (Cat. # 743977, RRID: AB_2741898) from BD Biosciences, His48-FITC (Cat. # 11057082, RRID: AB_465100) from ThermoFisher or His48-PE (Cat. # sc-19613PE, RRID: AB_627686) from SantaCruz Biotechnology, MHCII-FITC (Cat. # 11-0920-82, RRID: AB_465159) obtained from Thermo Fisher Scientific and CD163-PerCP (Cat. # NBP2-39099PCP, RRID: AB_2861402) from Novus Biologicals. Samples were acquired on a FACSCantoII (BD Biosciences) and analysed using FlowJo software (RRID: SCR_008520). At least 10,000 events were recorded. The flow cytometric analysis provided proportions of each peritoneal leukocyte sub-population (macrophages, neutrophils and monocytes, see the gating strategy presented in **Figure 1A**). The total number of leukocytes in the cavity was then calculated by combining FACS percentages and total cell numbers previously counted.

Rat peritoneal macrophages were separated and isolated using flow cytometry cell sorter (FACSAria III, BD Biosciences).

### *In Vitro* Cytotoxicity Test

Peritoneal leukocytes were obtained from untreated rats and added in culture in a 96-well plate (37°C, 2*10^5^ cells/well) for 24 hours to promote macrophage adhesion. After washing, adherent macrophages were exposed for 24 hours to 4 different doses of CNT-7 or CNT-T (20, 10, 5 and 3 µg/cm^2^). The luminescent signal (CellTiter-Glo^®^ Reagent, Cat. # G7570 from Promega), proportional to cellular ATP, was measured by luminometer after exposure. Each value is relative to the control condition (cells not exposed to CNT).

### Monocyte Depletion

Busulfan (Cat. # B2635-10G) from Sigma Aldrich was dissolved in Dimethyl sulfoxide (DMSO) Hybri-Max™ (Cat. # D2650) and injected at a dose of 15 mg/kg twice subcutaneously in rat, 3 days before and 3 days after intraperitoneal injection of CNT-7 or CNT-T (19). Rats were sacrificed 15 days after CNT injection.

### BioPlex Cytokine Analysis

Levels of soluble IL-1β, IL-6, IL-17A, GRO/KC, CCL3, CCL2 and M-CSF in peritoneal fluid supernatants obtained from the peritoneal lavages from untreated or CNT-treated rats were analysed using the Bio-Plex Pro™ Rat Cytokine Assay from 3 hours to 30 days. This multiplex assay was performed according to the manufacturer’s protocols (Cat. # 171K1001M, RRID: AB_2861403) from Bio-Rad.

### RNA Sequencing

Peritoneal macrophages from CNT-7 or CNT-T-treated rats sacrificed after 15 days, were separated and isolated using flow cytometry cell sorting (FACSAria III, BD Biosciences). The purity of the obtained macrophage cell preparations was routinely > 93 % as assessed by Diff-Quick staining. RNA was isolated using RNeasy mini kit (Cat. # 74104) from Qiagen. RNA quantity was measured with Qubit™ RNA HS Assay Kit (Cat. # Q32852) from Thermofisher. RNA integrity and quality were assessed using an Agilent Bioanalyzer using the Agilent RNA 6000 Nano kit (Cat. # 5067-1511). All RNA used had an RNA Integrity Number between 9 and 10. Libraries were prepared starting from 100 ng of total RNA using the Illumina^®^ TruSeq^®^ Stranded mRNA Sample Preparation Kits following the standard protocol. Libraries were sequenced using an Illumina HiSeq1500 platform. Approximately 50 million paired end reads (2x125bp) per sample were obtained. All sequencing data were analysed using the Automated Reproducible MOdular workflow for preprocessing and differential analysis of RNA-seq data (ARMOR) pipeline (20). In this pipeline, reads underwent a quality check using FastQC (RRID : SCR_014583) (21). Quantification and quality control results were summarized in a MultiQC report (RRID : SCR_014982) (22) before being mapped using Salmon (RRID : SCR_017036) (23) to the transcriptome index which was built using all Ensembl cDNA sequences obtained in the Rattus_norvegicus.Rnor_6.0.cdna.all.fa file (24). Then, estimated transcript abundances from Salmon were imported into R using the tximeta package (25) and analysed for differential gene expression with edgeR (RRID : SCR_012802) (26). Multiple testing correction of the p-value was performed within edgeR by the Benjamini-Hochberg procedure in order to control the false discovery rate (FDR) at a level of 0.10. Accordingly, genes associated with an adjusted p-values < 0.10 were considered significant and were therefore expected to include 10 % of false positive result. A downstream analysis was performed in order to interpret the differential expression results by considering a global biological context. In that context, pathway enrichment analysis was performed by using the WebGestaltR bioconductor package and the KEGG database (RRID : SCR_012773). Barcode gene enrichment plots were generated with limma bioconductor package (RRID : SCR_010943). The barcode plots are a visual representation of RNA-seq data where each vertical bar is a gene belonging to a specific pathway. The genes are ranked from left to right by increasing log-fold change (ratio of the gene expression level between CNT-7 and CNT-T) and the curve above the barcode plot is the relative local enrichment of the bars in the plot.

Both raw and processed RNA-seq data were deposited on the Gene Expression Omnibus (GEO) and made publicly available (GSE157487).

### BrdU Incorporation

After centrifugation of the peritoneal fluid from untreated and CNT-7 or CNT-T-treated rats (7 days), total peritoneal cells were seeded on polypropylene tubes and incubated for 24 h in RPMI 1640 Medium (Cat. # 21875-034) from Thermofisher supplemented with 10 % of decomplemented Fetal Bovine Serum (FBS) (Cat. # 10270-106) (Thermofisher) and 1 % antibiotic (Cat. # 15240-062) from Thermofisher. The BrdU was added to the culture (10 μM) 24 hours at 37 °C. 100 000 cells were stained with CD11b/c and HIS48 antibodies and fixed and permeabilized using the Cytofix/Cytoperm Buffer provided with the FITC BrdU Flow Kit (Cat. # 559619, RRID: AB_2617060) from BD Biosciences, according to the protocol. After permeabilization, cells were stained using anti-BrdU antibody provided with the kit. The samples were then analysed by flow cytometry.

### Ki-67 Staining

Peritoneal macrophages from untreated and CNT-7 or CNT-T-treated rats (7 days), were sorted out (CD11b/c and HIS48 staining, FACSAria III), fixed and permeabilized with a 3:1 ice-cold methanol-acetone mixture for 30 minutes. After the staining with anti-ki67 antibody (BD Pharmingen) (Cat. # 556026, RRID: AB_396302) samples were analysed by flow cytometry.

### qRT-PCR

qRT-PCR was performed on purified peritoneal macrophages from untreated or CNT-treated rats at (15 days). Total RNA extraction was performed using TriPure Isolation Reagent (Cat. # 11667165001) from Roche Diagnostics. Between 100 ng and 1 μg of RNA was reverse transcribed with MLV Reverse Transcriptase (Cat. # 28025013) from Thermofisher with 350 pmol random hexamers (Eurogentec, Seraing, Belgium) in a final volume of 25 μl. The resulting cDNA was used as template in subsequent polymerase chain reactions (PCR). Sequences of interest were amplified by PCR using the following forward primers from Invitrogen: TGCCTGTTTGGAAGGAGTATTGA (Mki67), CGGCTACCACATCCAAGGAA (h18sRNA) and reverse primers: TTTATAATAGTGCCATTTACTTGAGTTGGAT (Mki67), ATACGCTATTGGAGCTGGAATTACC (h18sRNA). PCR was performed with AmpliTaq Gold polymerase (Invitrogen) according to the manufacturer’s instructions with the following temperature program: 10 min 95 °C, (15 s 95 °C, 1 min 60 °C) ×40 cycles. Amplified DNA fragments were purified from a 2 % agarose gel using PCR clean-up Gel extraction kit(s) (Macherey-Nagel, Düren, Germany) and then serially diluted to serve as standards in real-time PCR. Reverse transcribed mRNAs were finally quantified by real-time PCR using SYBR Green technology on an ABI Prism 7000 Sequence Detection System (Applied Biosystems) according to the following program: 2 min 50 °C, 10 min 95 °C, (15 s 95 °C, 1 min 60 °C) ×40 cycles. To verify specific amplification, a dissociation curve was obtained by increasing temperatures up to 95 °C over a 20-minute period. Five μl of diluted cDNA or standards were amplified with 300 nM of the described primers using SYBR Green PCR Master Mix (Applied Biosystems) in a total volume of 25 μl. Results were calculated as a ratio of gene expression to the expression of the reference gene, RNA 18s.

### Immunohistochemistry

Omentum tissues from untreated and treated rats were harvested 1 hour after CNT-injection, fixed for 48h at 4 °C in 4 % paraformaldehyde and embedded in paraffin. Five‐µm sections were incubated with mouse anti‐rat CD68 antibody (Abcam monoclonal, Cat. # ab31630, RRID: AB_1141557) 90 minutes at RT, followed by 40‐min of incubation with secondary antibody donkey anti-mouse IgG (Cat. # 715035151, RRID: AB_2340771) from Jackson ImmunoReserch, coupled with HRP. The samples were then incubated for 10 minutes with AlexaFluor Tyramide 488 at RT. A counterstaining to detect the nuclei was performed with Hoechst44432 dye (Cat # 14533) from Sigma Aldrich. Stained slides were digitalized using a Pannoramic 250 FlashIII scanner (3DHistech) at x20 magnification. Whole tissue sections were then analysed using Visiopharm software (v2017.2). Tissue was first manually delineated at low magnification and large empty spaces and artifacts were automatically discarded. Cells were then detected at high resolution (20×) with a nuclear-based cell classification relying on the Hoechst staining. Following segmentation, post processing steps were applied to separate CD68-stained and unstained cells. The same parameters were kept constants for all slides. Results were expressed as percentage of stained cells.

### Statistics

Data were evaluated by one-way analysis of variance (ANOVA) using the Dunnett’s Multiple Comparison Test when appropriate. Statistical significance was considered at p < 0.05.

## Results

### Rapid Macrophage Disappearance Reaction and Repopulation After Injection of Mesotheliomagenic CNT-7

We first assessed the proportion and the number of macrophages in the peritoneal cavity after CNT injection. Wistar rats were intraperitoneally injected with 2 mg of CNT-7 or CNT-T (4). After sacrifice, the peritoneal fluid was collected, and the total peritoneal leukocytes were then counted (**Supplementary Figure 1**). The macrophage population was analysed by flow cytometry in peritoneal lavage (CD11bc^high^ and His48^dim^ cells, **Figure 1A**, frame a, among the other leukocyte populations frame b neutrophils and c monocytes). **Figures 1B–E** shows the proportion and the number of peritoneal macrophages accumulated at 0, 3 hours, 1, 3, 7, 15 and 30 days. Resident macrophages were depleted in the peritoneal cavity fluid from 3 hours to 3 days after treatment with both CNT in comparison with controls (**Figures 1B–E**), reflecting a macrophage disappearance reaction (MDR). This MDR was followed by a replenishment phase with a different macrophage repopulation according to the type of CNT. Indeed, macrophage regeneration (proportion and total counts) in CNT-7-treated rats was almost complete after 7 days (**Figures 1B–C**) in comparison to controls. The renewal reaction was slower (day 15, macrophage proportion) in CNT-T-treated rats and remains still incomplete after 30 days when considering macrophage number (**Figures 1D–E**). These data suggested that the macrophage replenishment phenomenon arising after early MDR is more rapid and pronounced in mesotheliomagenic response.

### Mesotheliomagenic CNT-7-Induced MDR Results From Cytotoxicity and Is Concomitant to Neutrophilic Inflammation

**Figure 2:**
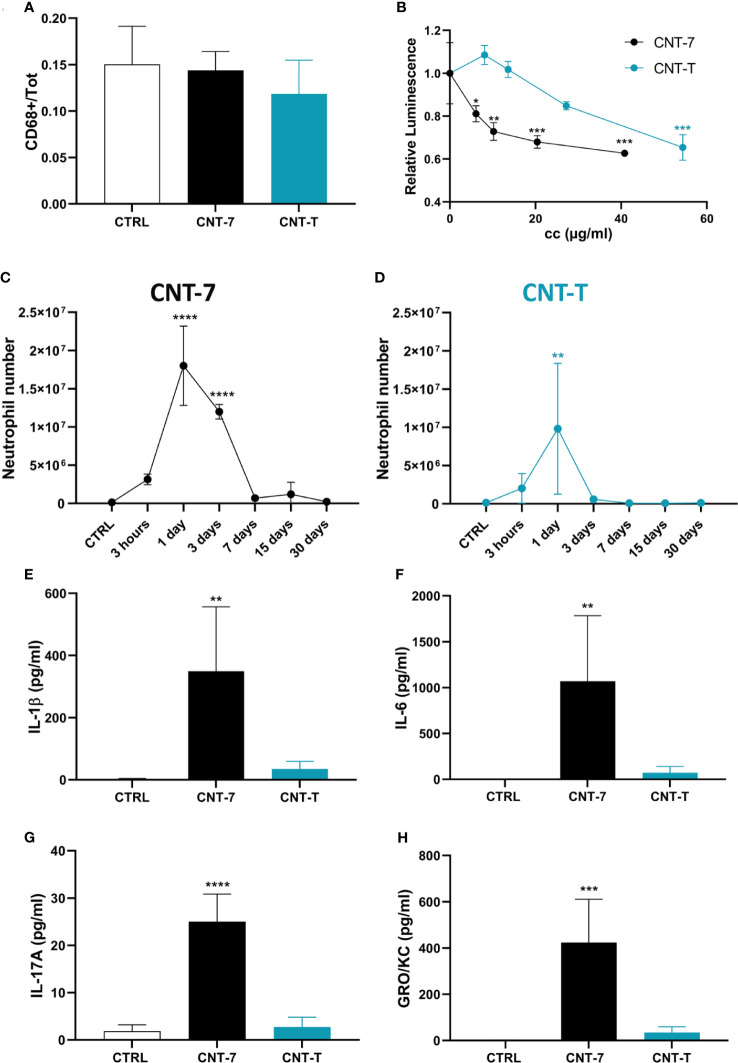
An early cytotoxicity but not a relocation in the omentum explains the CNT-induced disappearance reaction (MDR) and inflammatory responses. **(A)** Quantification of CD68-positive cells of the omentum of untreated and CNT-7 or CNT-T-treated rats (2 mg, I.P.) sacrificed after 1 hour. The immunofluorescent staining was performed with anti‐rat CD68 antibody. **(B)** Cellular ATP levels measured 24 hours after *in vitro* exposure of naïve peritoneal macrophages to 20, 10, 5 and 3 μg/ml of mesotheliomagenic CNT-7 or non-mesotheliomagenic CNT-T. ATP concentration was measured with CellTiter-Glo^®^ Luminescent Cell Viability Assay. Each point represents the mean of 3 replicates ± SD. **(C, D)** Number of peritoneal neutrophils (see Figure 1 A, frame b) in response to CNT-7 and CNT-T (2 mg, I.P.) (day 0-30). CTRL are untreated rats sacrificed at 3 hours. Each point represents the mean of 4 samples ± SD. **(E–H)** IL-1β, IL-6, IL-17A and GRO/KC released in untreated and CNT-7 or CNT-T-treated rat (2 mg, I.P.) peritoneal fluids at 3 hours were tested using Bio-Plex multiplex assay. The results were statistically analysed using ANOVA test followed by Dunnett’s test. (*) p = 0.0332, (**) p = 0.0021, (***) p = 0.0002, (****) p < 0.0001 indicates a statistically significant difference with controls.

The mechanism explaining CNT-induced MDR was investigated. We excluded a migration of macrophages from peritoneum to omentum. Indeed, no CD68-positive macrophage accumulation was observed in the omentum after CNT injection in comparison to control tissue (**Figure 2A** and **Supplementary Figure 2**). We next considered the possibility that the cytotoxic activity of CNT could explain macrophage disappearance. We exposed peritoneal macrophages from untreated rats to different doses of CNT *in vitro* and assessed cell viability by measuring ATP cellular contents. CNT-7 and CNT-T both exerted a dose-dependent cytotoxicity, with a higher effect at low doses for CNT-7 (**Figure 2B**), suggesting that the *in vivo* MDR resulted from the intrinsic cytotoxicity of CNT-7 and CNT-T to resident macrophages.

We also noticed that, after CNT-7, MDR and macrophage death were strongly related to an influx of neutrophils (**Figure 2C**; gating strategy see **Figure 1A**, frame b) and to a broad release of pyroptosis-associated IL-1β and cytokines (IL-6, IL-17A,GRO/KC and CCL-3) (**Figures 2E–H** and **Supplementary Figure 3**) in the peritoneal cavity. In contrast, limited inflammatory responses in time and amplitude were observed after CNT-T injection (**Figures 2D–H**). These data indicated that MDR is concomitant with a pyroptotic and inflammatory phase, which is faster and more pronounced for mesotheliomagenic CNT-7 than for non-mesotheliomagenic CNT-T.

### Mesotheliomagenic CNT-7 Specifically Induce MHCII^high^ Small Peritoneal Macrophage Expansion

We next studied the replenishment phase after MDR by characterizing the phenotype of regenerated macrophages 15 days after CNT-7 or CNT-T injection. For this purpose, we coupled RNA-sequencing data with flow cytometry analysis to characterize the peritoneal macrophage phenotype after regeneration. We compared the gene expression profile of newly accumulated macrophages with the already described MHCII^high^ SPM (Small Peritoneal Macrophages) and MHCII^low^ LPM (Large Peritoneal Macrophages) (13, 14, 27, 28).

**Figure 3:**
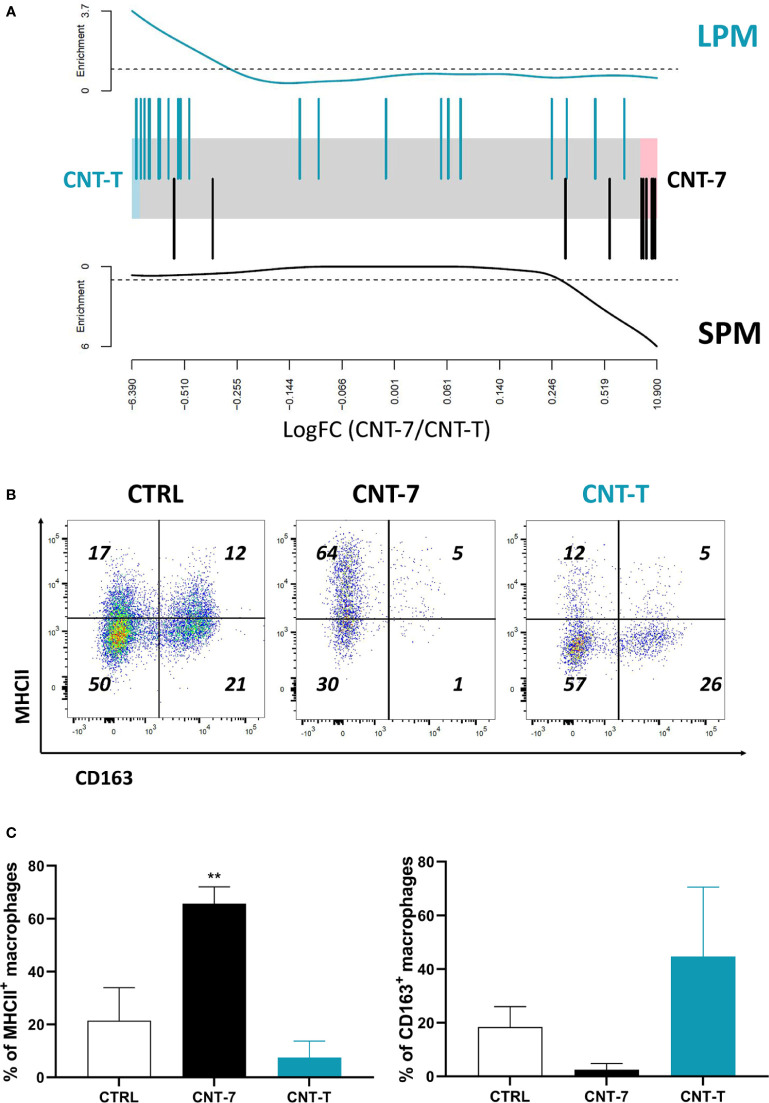
Mesotheliomagenic CNT-7 and non-mesotheliomagenic CNT-T exposure induces MHCII^high^ SPM and MHCII^low^ LPM macrophage development, respectively. **(A)** Barcode plot showing enrichment of MHCII^low^ (LPM-like) (in green, top panel) and MHCII^high^ (SPM-like) (in black, bottom panel) signatures in the CNT-7 *vs* CNT-T macrophage comparison. Macrophages were purified from the peritoneal cavity of rats treated with CNT-7 or CNT-T (2 mg I.P.) and sacrificed after 15 days. Black bars show up MHCII^high^ signature genes, blue bars show up MHCII^low^ signature genes. The worms show relative enrichment. **(B)** Flow cytometry dot plots (MHCII-CD163) of CD11bc^high^ and HIS48^dim^ macrophages from untreated and CNT-7- or CNT-T-treated Wistar rats (2 mg, I.P.) at 7 days. **(C)** Percentage of MHCII and CD163-positive macrophages from untreated or CNT-7/CNT-T-treated rats sacrificed 7 days after. Each bar represents the mean of 3 replicates ± SD. The results were statistically analysed using ANOVA test followed by Dunnett’s test. (**) p = 0.0021 indicates a statistically significant difference with controls.

As illustrated in the barcode plot (**Figure 3A**, bottom panel) and heatmap (**Supplementary Figure 4A**), there was an enrichment of MHCII^high^ SPM gene signature in the CNT-7 *versus* CNT-T macrophages. In agreement with these results, we observed that macrophages recruited after CNT-7 markedly expressed MHC-II (but not CD163) in comparison to controls and CNT-T treatment (**Figures 3B–C**). Our data thus suggested that mesotheliomagenic CNT-7 specifically regenerated macrophages with an MHC-II^high^ SPM-like phenotype. In contrast, macrophages collected from CNT-T-treated rats expressed genes related to the MHCII^low^ LPM phenotype (Barcode data, **Figure 3A**, top panel and heatmap (**Supplementary Figure 4B**). As appreciated by flow cytometry, these macrophages were mainly CD163-positive and MHC-II-negative, and thus corresponded to the macrophage phenotype in homeostatic conditions (**Figures 3B–C**).

### MHCII^high^ and MHCII^low^ Macrophage Replenishment During CNT Responses Depend on Monocyte-Lineage Progenitors

**Figure 4:**
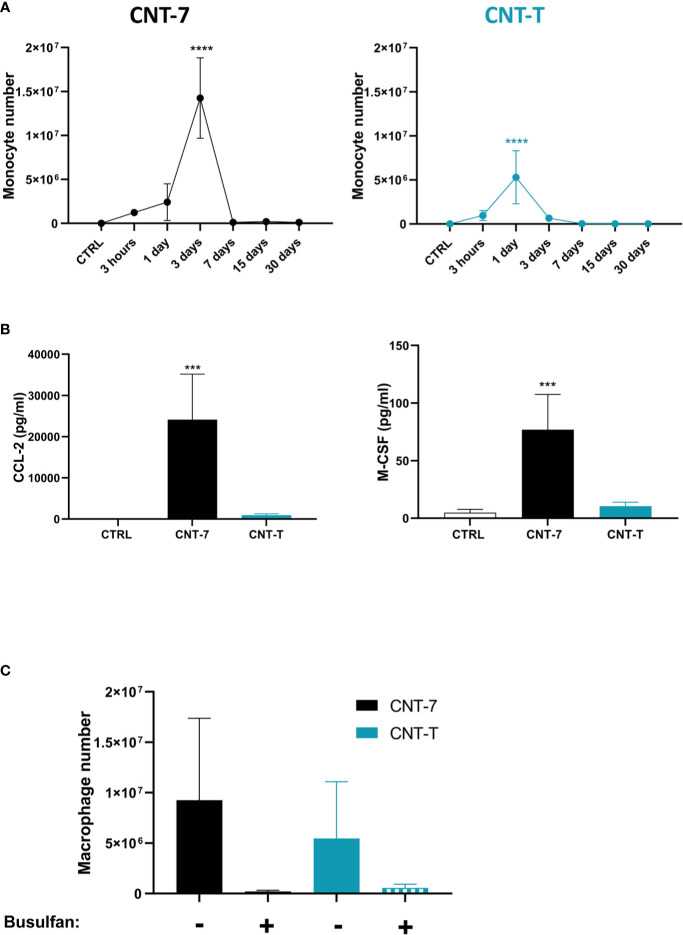
Macrophage regeneration after CNT-7 treatment depends on blood monocytes. **(A)** Number of peritoneal monocytes in response to mesotheliomagenic CNT-7 and non-mesotheliomagenic CNT-T (2 mg, I.P.) (day 0-30). CTRL are untreated rats sacrificed at 3 hours. Each point represents the mean of 4 samples ± SD. **(B)** CCL2 and M-CSF released at 3 hours in untreated and CNT-7 and CNT-T-treated rat (2 mg, I.P.) peritoneal fluid were tested using Bio-Plex multiplex assay. M-CSF and CCL2 were found to be abundantly produced by CNT-7-treated rats. **(C)** Percentage of peritoneal macrophages (CD11bc^high^ HIS48^dim^) after S.C. injection of double dose of Busulfan (15 mg/kg) before and after injection of CNT-7 and CNT-T. Rats were sacrificed 15 days after CNT injection. Each bar represents the mean of 3 replicates ± SD. The results were statistically analysed using ANOVA test followed by Dunnett’s test. (***) p = 0.0002, (****) p < 0.0001 indicates a statistically significant difference with controls.

Our phenotypic data suggested that regenerated MHCII^high^ SPM macrophages after mesotheliomagenic CNT-7 derived from monocytic progenitors. We assessed monocyte composition of peritoneal lavages and found that CNT-7 induced a clear accumulation of monocytes (CD11b/c^int^ His48^high^, gating strategy see **Figure 1A**, frame C) starting at 3 hours to reach a peak at 3 days, just before macrophage repopulation (**Figure 4A**). This reaction was less important and limited to day 1 after CNT-T (**Figure 4A**). Moreover, high levels of CCL2 and M-CSF, essential for monocyte chemo-attraction, survival and differentiation were detected at 3 hours in peritoneal fluid of CNT-7-treated rats in comparison to CNT-T and untreated rats (**Figure 4B** and **Supplementary Figure 5**). These data additionally supported a monocytic ontogeny for MHCII^high^ macrophages. Finally, we also depleted the source of monocytes by depleting the bone marrow progenitors with Busulfan and appreciated macrophage regeneration in CNT-treated rats. Peritoneal macrophage replenishment was completely abrogated in monocyte-depleted animals treated with CNT-7 (**Figure 4C**). While monocytes and related cytokines were not massively accumulated after CNT-T treatment (**Figures 4A, B**), we also observed that monocyte-depletion affected macrophage replenishment (**Figure 4C**). Altogether, these results demonstrated that the replenishment of the peritoneal cavity by MHCII^high^ SPM and to a lesser extent MHCII^low^ LPM relies on a monocytic source after mesotheliomagenic CNT-7- and CNT-T-induced MDR.

### Macrophages Replenishing the Peritoneal Cavity After Non-Mesotheliomagenic CNT-T Are Self-Renewing

**Figure 5:**
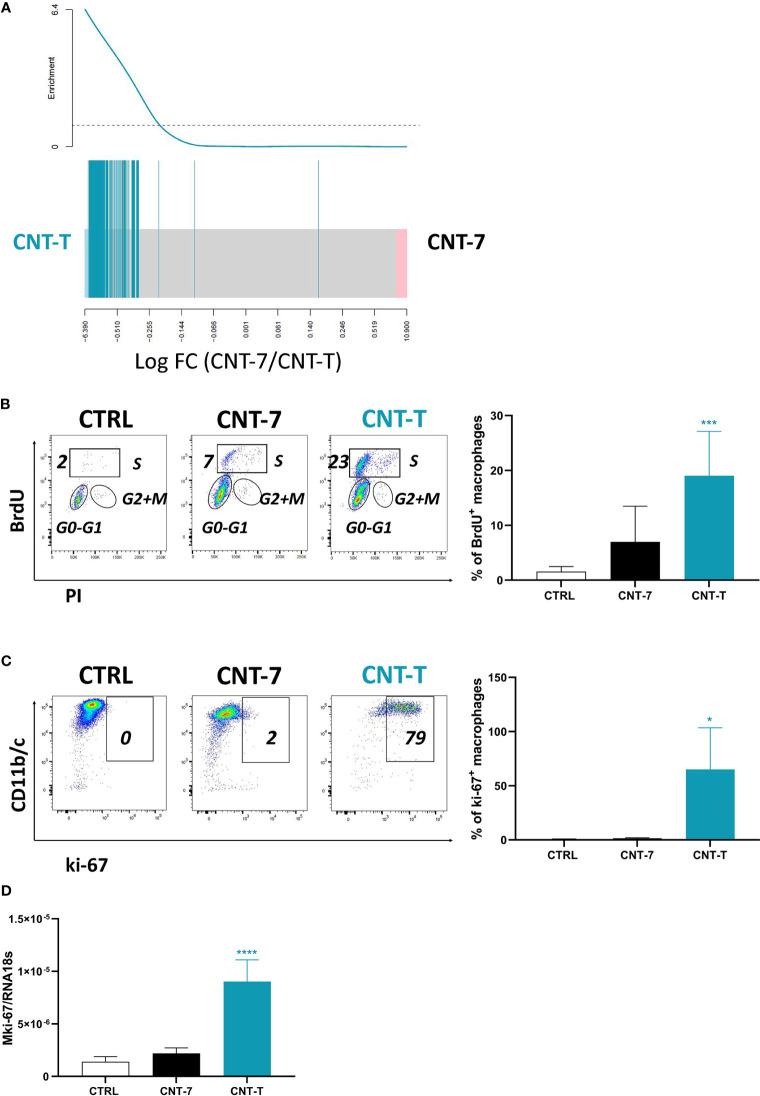
Macrophages repopulating the peritoneal cavity after non-mesotheliomagenic CNT-T treatment are self-renewing. **(A)** Barcode plot showing enrichment of cell cycle related signature belonging to the “Cell Cycle/Mitotic” pathway R-RNO-69278 from Reactome database in the CNT-7 *vs* CNT-T macrophage comparison (respectively in black and green). Macrophages were purified from the rat peritoneal cavity after CNT-7 or CNT-T treatment (2 mg I.P.) at day 15. The worms show relative enrichment. **(B)** Flow cytometry dot plots show the proportions of peritoneal macrophages in S phase (BrdU-positive) of untreated Wistar rats or rats injected (2 mg I.P.) with CNT-7 or CNT-T and sacrificed at 7 days. Graph of BrdU-positive macrophages: each bar represents mean ± SD of 6 observations. **(C)** Flow cytometry dot plots show the proportions of peritoneal macrophages ki-67-positive from untreated or CNT-7/CNT-T treated rats sacrificed at 7 days. The graph represents the ki-67-positive macrophages from untreated rats and CNT-7 or CNT-T-treated rats. Each bar represents mean ± SD of 6 observations. **(D)** mki-67 from macrophages purified from untreated and CNT-treated rats (at 15 days) was quantified by qRT-PCR. Values are means ± SD of 4 animals. The results were statistically analysed using ANOVA test followed by Dunnett’s test. (*) p = 0.0332, (***) p = 0.0002, (****) p < 0.0001 indicates a statistically significant difference with controls.

In comparison to monocyte-derived MHCII^high^ SPM macrophages, RNA-seq data revealed that several genes related to cell cycle and mitosis were specifically and strongly expressed by MHCII^low^ LPM macrophages after CNT-T (**Figure 5A**). To confirm their proliferative ability, we next studied BrdU incorporation and ki-67 expression. Based on BrdU data, MHCII^low^ macrophages from CNT-T-treated rats proliferated more than macrophages from untreated and CNT-7-treated rats (Flow cytometry dot plot and bar graph, **Figure 5B**). Similar data were obtained by analysing the expression of ki-67. MHCII^low^ CNT-T macrophages expressed more ki-67 transcripts (qRT-PCR, **Figure 5D**) and protein (Flow cytometry dot plot and bar graph, **Figure 5C**) than macrophages from untreated- or CNT-7-treated rats. Altogether, these results indicate that self-proliferating MHCII^low^ LPM become the prevalent macrophage subset in response to CNT-T in contrast to CNT-7.

## Discussion

CNT are attractive materials with unique physical and chemical properties, including tensile strength, stiffness and good electrical conductivity which are currently used in engineering and material sciences for rechargeable batteries, automotive parts, sporting goods and water filters (29). Experimental studies in rodents demonstrated that the long and straight CNT-Mitsui-7 can induce mesothelioma like asbestos fibres, and experts classified CNT-7 in the IARC Group 2b, i.e. as possibly carcinogenic for humans (5). The numerous applications of other CNT justify the need of carefully characterizing the mesotheliomagenic potential (30, 31).

Like for asbestos, the pathogenicity of CNT is linked to their shape and geometry (3). In rats, more sensitive than mice to mesothelioma development, a single intraperitoneal injection of CNT with a straight and acicular shape (CNT-7) induced peritoneal mesothelioma with a 100 % of incidence after 52 weeks. On the other side, tangled CNT (CNT-T) did not induce peritoneal mesothelioma in long-term studies (3 years) (17, 32–34).

In homeostatic conditions the peritoneal cavity is populated by resident macrophages, free-floating GATA6^+^ cells able to proliferate locally without the contribution of bone marrow-derived monocytes (35). Inflammatory stimuli trigger a phenomenon called Macrophage Disappearance Reaction (MDR), during which resident macrophages become irretrievable from the lavage of the serous cavity. This phenomenon was already observed after different inflammatory insults (14, 16, 27, 35), but is still incompletely understood. MDR can be associated with cell death, adhesion to neighbouring tissues or migration to the draining lymph nodes or the omentum (14, 27). MDR is a strategy to face and annihilate the infection by which macrophages, under the control of GATA6, move from the peritoneum to the closest tissues in order to alert the immune system. At the same time, they express factor V of the coagulation cascade to promote clot formation in order to trap free-floating bacteria or foreign particles and facilitate their phagocytosis (36). Here, we report a clear disappearance reaction in the rat peritoneal cavity only 3 hours after the injection of both kinds of CNTs (CNT-T and CNT-7). Interestingly, MDR was not linked to a migration to neighbouring tissue but was caused by the cytotoxic activity of CNT to peritoneal resident macrophages.

In steady state conditions, even if the peritoneal cavity is easily accessible from blood vessels, blood monocytes do not contribute to the existing pool of resident macrophages (37). However, when resident macrophages are depleted during inflammatory stimuli, macrophages from circulating monocytes or self-proliferating resident macrophages represent the two competing mechanisms to achieve the repopulation of empty cavities. This phenomenon is called ‘niche model’ and occurs when the compartment is accessible (no physical barriers), available (not occupied by other macrophages) and without other competitive progenitors (37). The degree of the replacement depends on the magnitude of inflammation. In case of severe inflammation all the resident macrophages are depleted and then replaced by bone marrow-derived monocytes which acquire a mature resident phenotype characterized by an accentuated expression of MHCII complex (38). A mild inflammation, conversely, preventing a complete loss of resident cells, triggers a lower recruitment of monocytes which differentiate into highly proliferating, immature cells (38). As debated below, our data are in complete accordance with the niche and inflammation magnitude concepts and support their utilities in particle toxicology and mesothelioma fields.

The regeneration of macrophages by monocytes is orchestrated by dying macrophages releasing master inflammatory mediators (mainly members of the IL-1 and TNF families). These inflammatory molecules temporarily trigger macrophage niche (epithelial, endothelial and stromal cells) to release monocyte-recruiting (CCL2 and adhesion molecules) and differentiating (CSF family) factors that result in monocyte engraftment and differentiation (39). The exact growth factors orchestrating monocyte differentiation or macrophage proliferation are still debated but the most accepted hypothesis is that the expression of soluble or membrane forms of M-CSF, IL-34 or GM-CSF and the complex association of their corresponding receptors are crucial to fill the empty niches and finalize the macrophage repopulation (15).

Our data support the new concept that the regeneration of macrophages during mesotheliomagenic responses to CNT-7 is exclusively tributary of the monocyte pool. The initial depletion depends on the cytotoxic activity of the particles to macrophages, and the intensity and duration of the inflammation, which is related to the type of macrophage death. Indeed, the rigid, needle-like shape of CNT-7 is responsible for a pronounced inflammatory reaction initiated by pyroptosis/inflammasome mediated-cell death (IL-1β activation and release) and release of inflammatory cytokines (IL-6, IL-17A, CCL3, GRO/KC) involved in neutrophil recruitment. The cytotoxic and inflammatory activity of CNT-7 in turn triggers the recolonization of the niche by inflammatory monocytes which differentiate into pathogenic macrophages.

The dynamic of macrophage replenishment after mesotheliomagenic CNT-7-induced depletion could rely on the CCL2 and M-CSF axis (**Figure 4B**). CCL2 (MCP-1) is a chemokine regulating monocyte infiltration in tissues. The major source and the main cell target of CCL2 is the monocyte/macrophage lineage. CCR2^high^ monocyte-derived macrophages typically infiltrate cancer promoting tumor growth, immunosuppression, angiogenesis, and cancer cell dissemination leading to a bad prognosis (40). Anti-CCL2 neutralizing antibodies reduce mesothelioma growth and tumor volume, switching the polarization of tumor infiltrating macrophages to a more anti-tumor phenotype (41). M-CSF controls the production, differentiation, survival and functions of macrophages, monocytes and bone marrow progenitors. Like CCL-2, M-CSF is involved in monocyte chemoattraction and promotes monocyte survival (42). M-CSFR (also known as CSF1-R) inhibitors induce the depletion of tumor infiltrating macrophages or their polarization towards a pro-inflammatory phenotype, with beneficial effects for mesothelioma regression (43, 44).

After the macrophage disappearance reaction, monocytes from CNT-7-treated rats engrafted the empty peritoneal cavity and differentiated into resident cells, possessing a unique MHCII^high^ SPM phenotype. Serous cavities physiologically comprise distinct macrophages subsets. Monocyte-derived MHCII^high^ macrophages are characterized by highly phagocytic activity and constitutively producing RELM-alpha, a cytokine signature of alternatively activated macrophages (M2) that controls the CD4^+^ T cell response during inflammatory events and promotes tissue repair process (45). It was recently discovered that MHCII^high^ macrophages present the antigens to naïve CD4^+^ T cells, thanks to DNAM-1, an immunoreceptor, co-immunostimulatory signal which binds its counterpart (CD155 ligand) on CD4^+^ T cells (46). These data are in accordance with our previous results showing that only mesotheliomagenic particle injection induced an accumulation of monocytes and macrophages possessing the ability to limit T cell functions (4). This alternative polarization of monocyte-derived macrophages after CNT-7, is possibly kept until the installation of cancer, contributing to prevention of T cell killer activity, as widely described in the literature (11).

We showed that the replenishment of the cavity after non mesotheliomagenic CNT-T is organised by monocyte-derived MHCII^low^ CD163^+^ macrophages. MHCII^low^ macrophages are maintained through local self-renewal in naïve conditions (47). They express GATA6, important for the proliferation, survival and physiologic metabolic activity of macrophages. Moreover, these cells represent an important reservoir of mature macrophages, which can be mobilized to repair injured tissues and orchestrate return to homeostasis (47). We demonstrated that MHCII^low^ macrophages presenting the characteristics of proliferating and self-renewing cells also accumulate after CNT-T. At the same time, these proliferating macrophages partially depend on hematopoietic progenitors since they are not completely replenished after monocyte depletion (**Figure 4C**). These findings agree with those published by Louwe and coauthors (38). They demonstrated that mild inflammation induced the recruitment of long-lived monocyte-derived macrophages joining the remaining resident cells in the peritoneal cavity. The competition for the niche signals between resident and newly arrived macrophages forced the latter to acquire a highly proliferative and immature GATA6^low^ MHCII^high^ phenotype which progressively differentiate into a GATA6^high^ MHCII^low^ mature state. In agreement with these observations, Cain and coworkers (48) showed that after resident cell depletion *via* chemical or radioactive treatment, circulating monocytes differentiate first into F4/80^low^ MHCII^high^ macrophages and later into F4/80^high^ MHCII^low^ proliferating macrophages. This represents the physiological process allowing to restore the reservoir of resident cells with bone marrow derived circulating monocytes after depletion. We thus propose that MHCII^low^ macrophages derive at very early time point from MHCII^high^ macrophages. Then, MHCII^low^ macrophages proliferate and reconstitute the peritoneal macrophage stock by proliferation.

**Figure 6:**
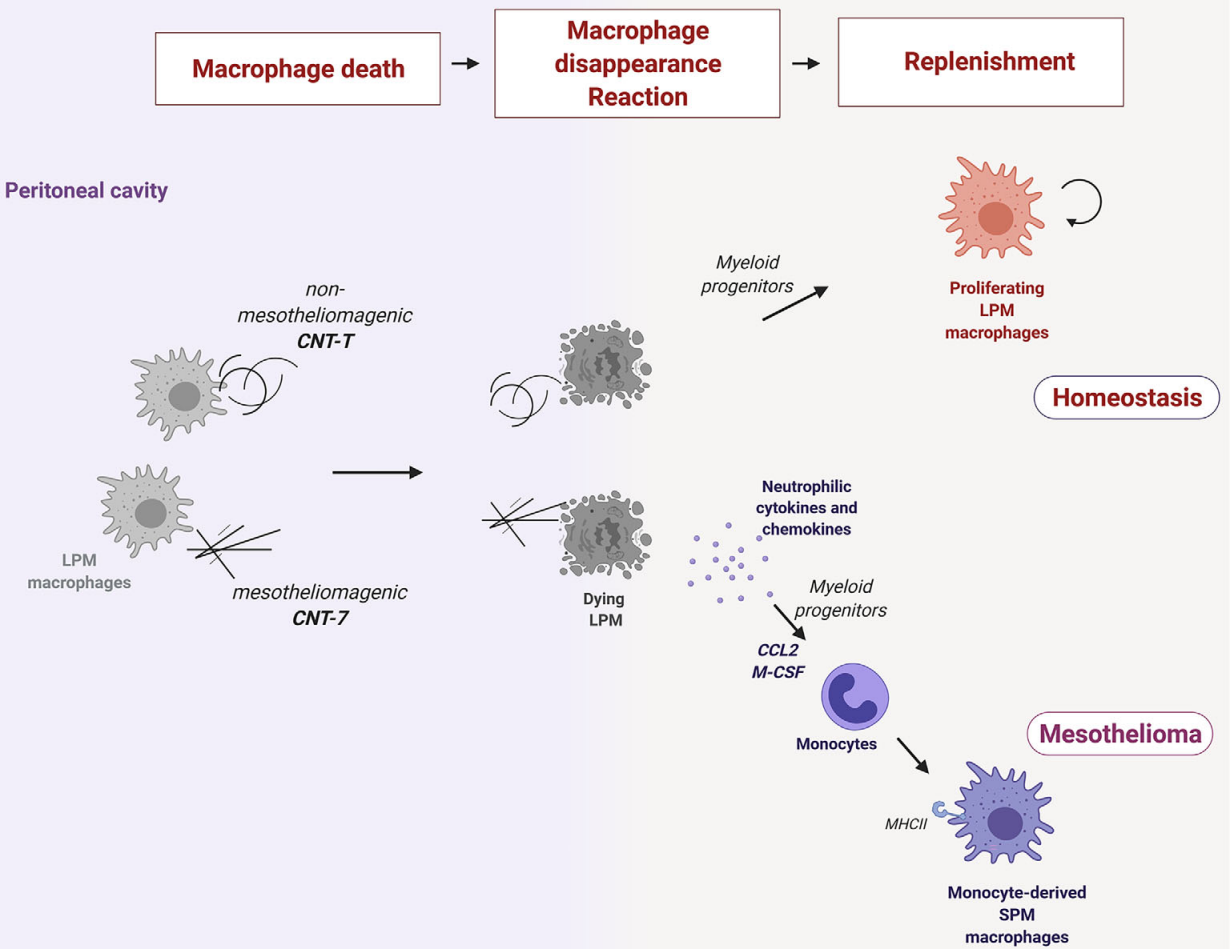
Resident macrophages phagocytize needle-like mesotheliomagenic particles (CNT-7), die rapidly (macrophage disappearance reaction or MDR) and release pyroptosis-related inflammatory cytokines (IL-1β, IL-6, and IL-17A) and chemokines (GRO/KC) to recruit neutrophils and alert the immune system. Released M-CSF and CCL2 chemokines also recruit bone marrow-derived monocytes, which then differentiate into MHCII^high^ small peritoneal macrophage (SPM-like) to replenish the empty macrophage niche. These monocyte-derived macrophages after CNT-7 may contribute to mesothelioma by preventing T cell killer activity. A MDR is also observed after phagocytosis of non-mesotheliomagenic tangled particles (CNT-T) but in a non-inflammatory environment. In this case, the replenishment of the cavity relies firstly on myeloid monocyte differentiation and later on proliferating MHCII^low^ large peritoneal macrophages (LPM).

Altogether, our findings suggest that the subtype of macrophages replenishing the peritoneal cavity during the early responses to particles is a key element in mesothelioma development (**Figure 6**). Pathogenic needle-like CNT are highly cytotoxic for resident macrophages in the peritoneal cavity. This cytotoxicity induces the release of pyroptotic-related inflammatory cytokines and chemokines, which, in turn, together with the need to repopulate the empty niche, orchestrate early immune responses characterized by the recruitment of monocytes that differentiate later into MHCII^high^ SPM. MHCII^high^ SPM macrophages express the signature of an alternative polarized phenotype (like RELM-alpha and DNAM-1) which characterize also Tumor associated Macrophages, infiltrating mesothelioma and limiting T cell anticancer activity. In contrast, the macrophage repopulation after non-mesotheliomagenic CNT is caused by the proliferation of newly differentiated MHCII^low^ macrophages from recruited monocytes. These proliferating macrophages re-colonize the peritoneum and can restore the normal equilibrium disrupted after particle-induced depletion without deploying inflammation.

These findings may open a horizon for a new therapeutic strategy for mesothelioma based on reprogramming of MHCII^high^ macrophages towards a MHCII^low^ profile and the reactivation of their cell cycle to revert their functions into non-pathogenic cells. Moreover, the regeneration and the niche model proposed in this study validate the interests for *in vivo* bioassays assessing MDR and macrophage repopulation for anticipating the hazardous potential of a new particle or fibre sample.

## Data Availability Statement

Both raw and processed RNA-seq data were deposited on the Gene Expression Omnibus (GEO) and made publicly available (GSE157487).

## Ethics Statement

The animal study was reviewed and approved by Comité d’Ethique pour l’Expérimentation Animale, Secteur des Sciences de la Santé, Brussels, Belgium (No LA1230312).

## Author Contributions

MO, FH, DB, BB, JA, and DL were involved in the conception and design of the experiments and data analysis. MO and FH wrote the manuscript. MO, MP-P, SI, and MB performed the experiments. All authors contributed to the article and approved the submitted version.

## Funding

This work was funded by the Actions de Recherche Concertées, Fédération Wallonie‐Bruxelles (ARC 19/24‐098, CYTAID), Fondation Contre le Cancer (2019‐219), Fonds de la Recherche Scientifique (FNRS, PDR T.0119.13), ANSES (Agence nationale française de sécurité sanitaire de l’alimentation, de l’environnement et du travail, MacFibOsis) and European Commission under H2020 project (Contract no. 874707, Eximious). FH is a Senior Research Associate with the FNRS, Belgium.

## Conflict of Interest

The authors declare that the research was conducted in the absence of any commercial or financial relationships that could be construed as a potential conflict of interest.

## Supplementary Material

Supplementary Figure 1 Total number of peritoneal leukocytes after exposure to mesotheliomagenic CNT-7 and non-mesotheliomagenic CNT-T. The total number of leukocytes in rat peritoneal cavity was counted on Burker chamber. Peritoneal fluids were collected from untreated rats (CTRL) and from rats treated with CNT-7/CNT-T at 3 hours and 1, 3, 7, 15 and 30 days. Each point represents the mean of 4 samples ± SD. The results were statistically analysed using one-way ANOVA test followed by Dunnett’s test. (*) p = 0.0332, (****) p < 0.0001 indicates a statistically significant difference with controls.

Supplementary Figure 2 Caption. Immunofluorescence images of CD68^pos^ macrophages in the omentum harvested from untreated **(A)** and CNT-7 **(B)** or CNT-T **(C)** treated rats at 1 hour. The green colour depicts CD68 glycoprotein while the blue colour in the images depicts the nucleus, stained with Hoechst44432 dye. Representative images are shown. Magnification, 40X.

Supplementary Figure 3 Levels of pro-inflammatory cytokines and chemokines in the peritoneal fluids after mesotheliomagenic CNT-7 and non-mesotheliomagenic CNT-T. **(A)** IL-1β, **(B)** IL-6, **(C)** IL-17A, **(D)** GRO/KC and **(E)** CCL-3 released in untreated (CTRL) and CNT-7 or CNT-T-treated rat (2 mg, I.P.) peritoneal fluids at 3 hours and 1, 3, 7, 15 and 30 days after injection were tested using Bio-Plex multiplex assay. Each point represents the mean of 4 samples ± SD. The results were statistically analysed using ANOVA test followed by Dunnett’s test. (*) p = 0.0332, (***) p = 0.0002, (****) p < 0.0001 indicates a statistically significant difference with controls.

Supplementary Figure 4 SPM and LPM phenotypes in peritoneal macrophages from mesotheliomagenic CNT-7- and non-mesotheliomagenic CNT-T-treated rats. **(A)** Heatmap of RNA-Seq expression z-score computed from the logcpm of a list of selected SPM genes in CNT-7 and CNT-T macrophages. Each row represents a gene characterizing the SPM profile, and each column represents a sample of macrophages from CNT-7 (1-4) or CNT-T (5-8) injected rat. **(B)** Heat map of RNA-Seq expression z-score computed from the logcpm of a list of selected LPM genes in CNT-7 and CNT-T macrophages. Each row represents a gene characterizing the LPM profile, and each column represents a sample of macrophages from CNT-7 (1-4) or CNT-T injected rat (5-8).

Supplementary Figure 5 Levels of monocytes-related cytokines in the peritoneal fluids after mesotheliomagenic CNT-7 and non-mesotheliomagenic CNT-T. **(A)** CCL-2 and **(B)** M-CSF released in untreated (CTRL) and CNT-7 or CNT-T-treated rat (2 mg, I.P.) peritoneal fluids at 3 hours and 1, 3, 7, 15 and 30 days after injection were tested using Bio-Plex multiplex assay. Each point represents the mean of 4 samples ± SD. The results were statistically analysed using ANOVA test followed by Dunnett’s test. (*) p = 0.0332, (****) p < 0.0001 indicates a statistically significant difference with controls.
