# Supplementary figures and images for: Monocytic Ontogeny of Regenerated Macrophages Characterizes the Mesotheliomagenic Responses to Carbon Nanotubes
Source: Front Immunol. 2021 Jun 14;12:666107. doi: 10.3389/fimmu.2021.666107 (PMC8236701; doi:10.3389/fimmu.2021.666107)

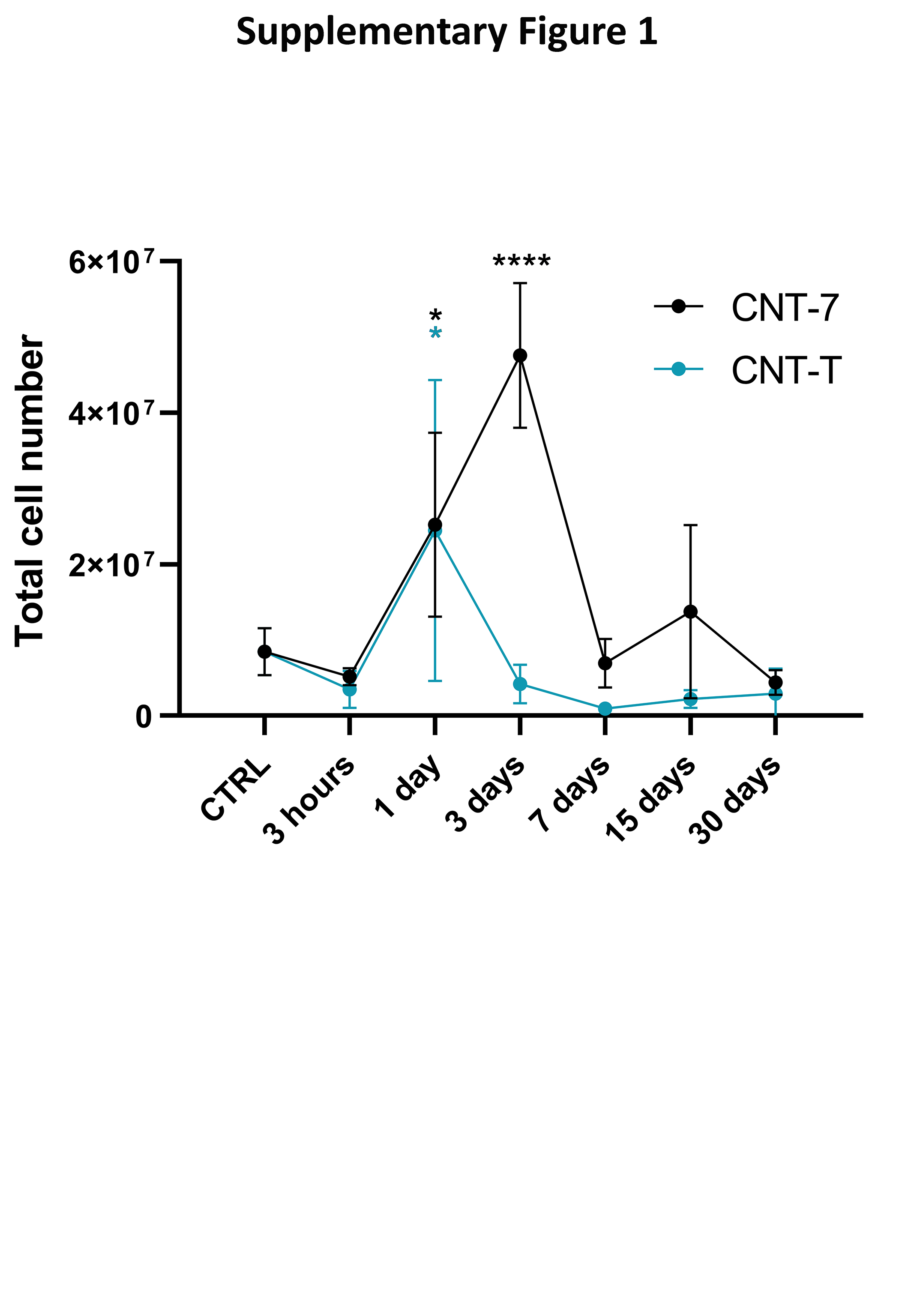

Supplement: Supplementary Figure 1 — Total number of peritoneal leukocytes after exposure to mesotheliomagenic CNT-7 and non-mesotheliomagenic CNT-T. The total number of leukocytes in rat peritoneal cavity was counted on Burker chamber. Peritoneal fluids were collected from untreated rats (CTRL) and from rats treated with CNT-7/CNT-T at 3 hours and 1, 3, 7, 15 and 30 days. Each point represents the mean of 4 samples ± SD. The results were statistically analysed using one-way ANOVA test followed by Dunnett’s test. (*) p = 0.0332, (****) p < 0.0001 indicates a statistically significant difference with controls. [file Image_1.tif]

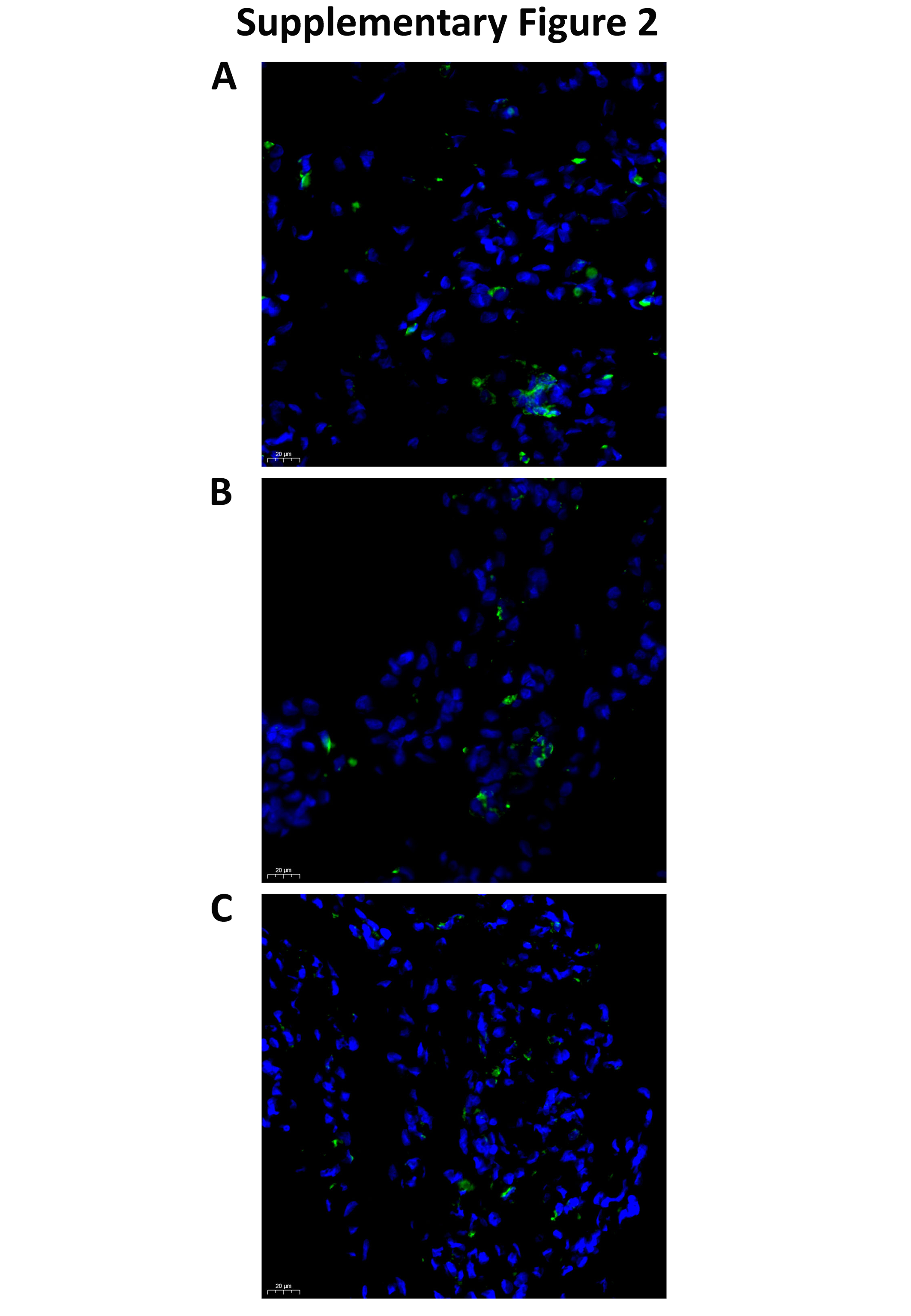

Supplement: Supplementary Figure 2 — Caption. Immunofluorescence images of CD68pos macrophages in the omentum harvested from untreated (A) and CNT-7 (B) or CNT-T (C) treated rats at 1 hour. The green colour depicts CD68 glycoprotein while the blue colour in the images depicts the nucleus, stained with Hoechst44432 dye. Representative images are shown. Magnification, 40X. [file Image_2.tif]

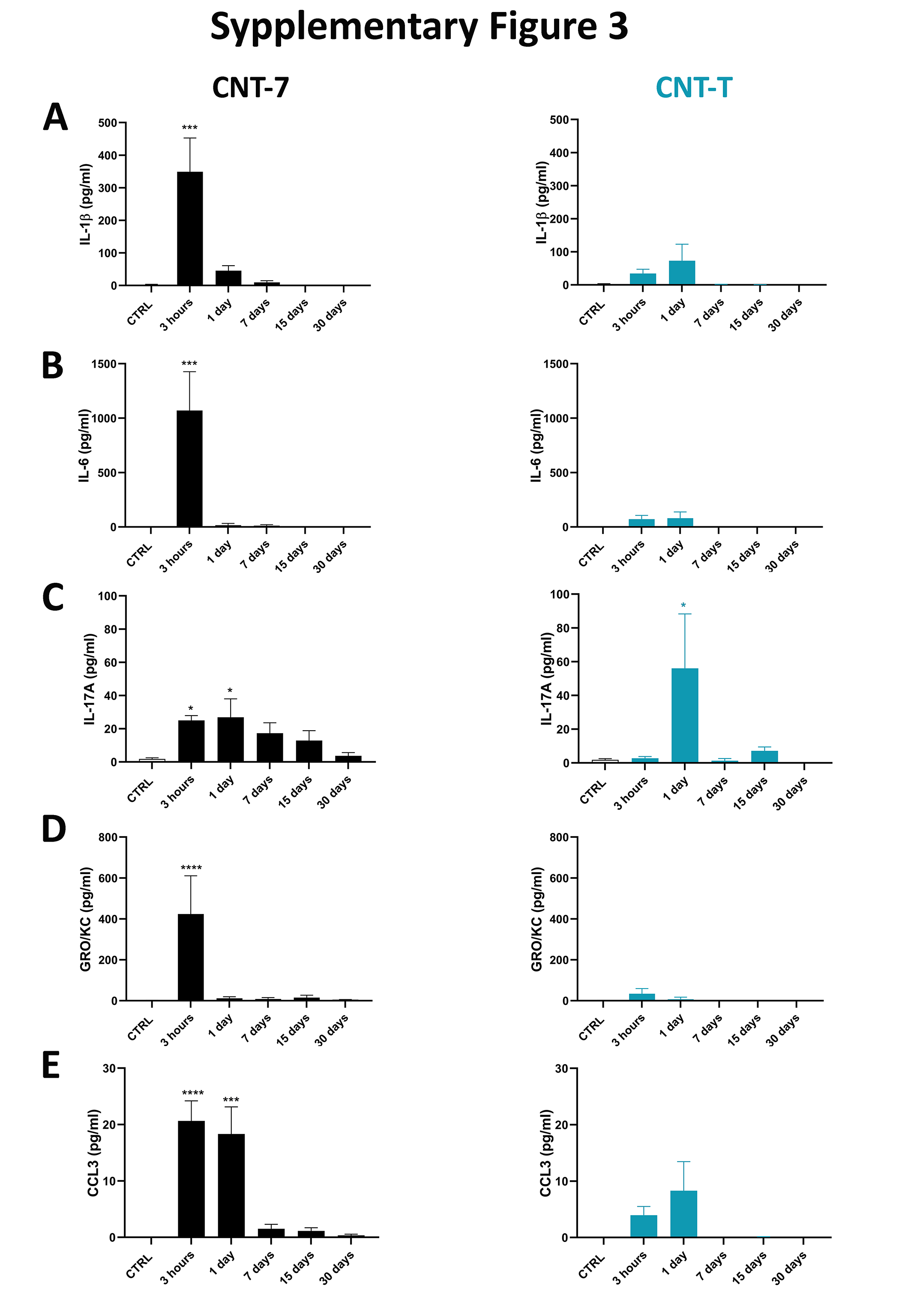

Supplement: Supplementary Figure 3 — Levels of pro-inflammatory cytokines and chemokines in the peritoneal fluids after mesotheliomagenic CNT-7 and non-mesotheliomagenic CNT-T. (A) IL-1β, (B) IL-6, (C) IL-17A, (D) GRO/KC and (E) CCL-3 released in untreated (CTRL) and CNT-7 or CNT-T-treated rat (2 mg, I.P.) peritoneal fluids at 3 hours and 1, 3, 7, 15 and 30 days after injection were tested using Bio-Plex multiplex assay. Each point represents the mean of 4 samples ± SD. The results were statistically analysed using ANOVA test followed by Dunnett’s test. (*) p = 0.0332, (***) p = 0.0002, (****) p < 0.0001 indicates a statistically significant difference with controls. [file Image_3.tif]

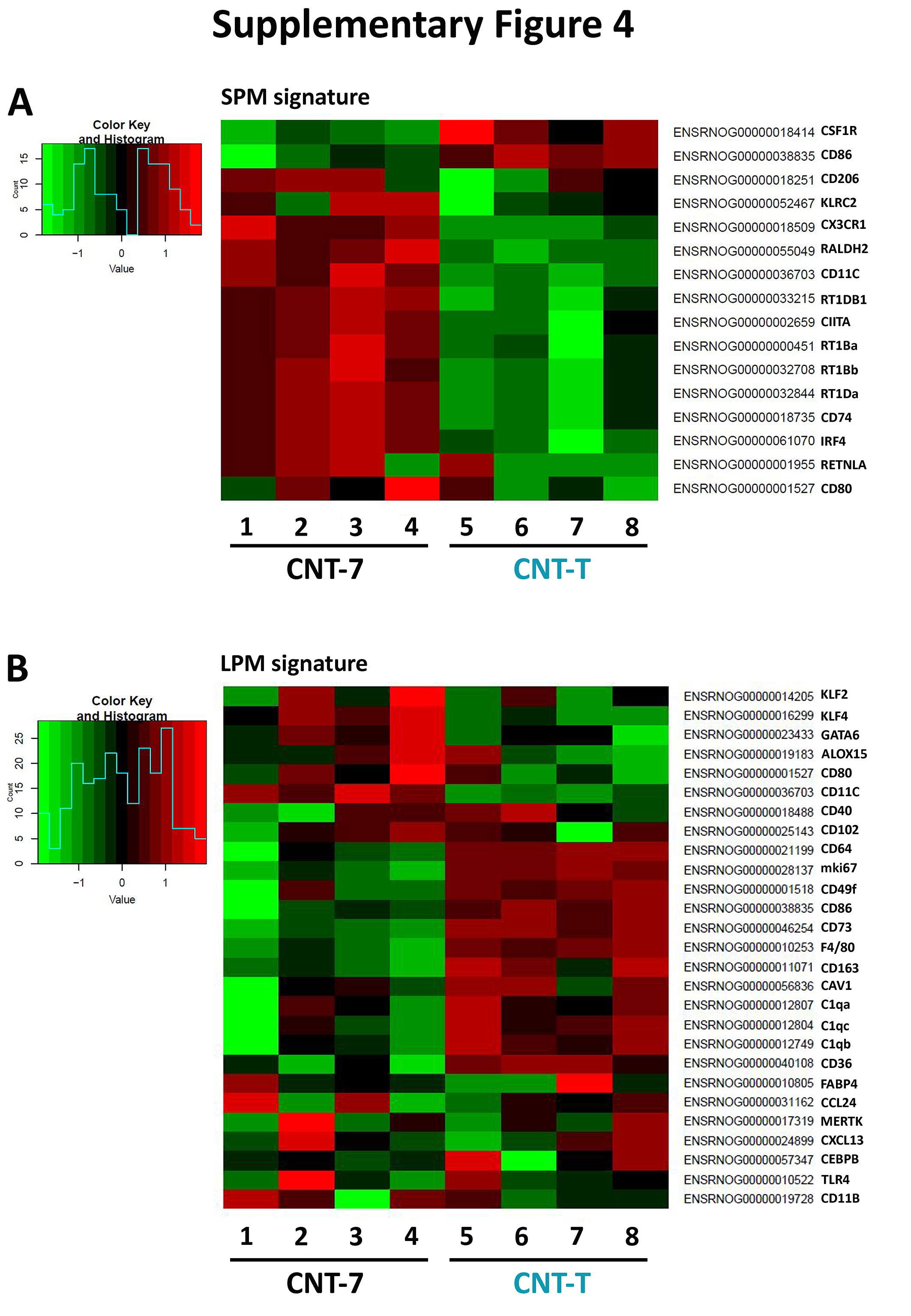

Supplement: Supplementary Figure 4 — SPM and LPM phenotypes in peritoneal macrophages from mesotheliomagenic CNT-7- and non-mesotheliomagenic CNT-T-treated rats. (A) Heatmap of RNA-Seq expression z-score computed from the logcpm of a list of selected SPM genes in CNT-7 and CNT-T macrophages. Each row represents a gene characterizing the SPM profile, and each column represents a sample of macrophages from CNT-7 (1-4) or CNT-T (5-8) injected rat. (B) Heat map of RNA-Seq expression z-score computed from the logcpm of a list of selected LPM genes in CNT-7 and CNT-T macrophages. Each row represents a gene characterizing the LPM profile, and each column represents a sample of macrophages from CNT-7 (1-4) or CNT-T injected rat (5-8). [file Image_4.tif]

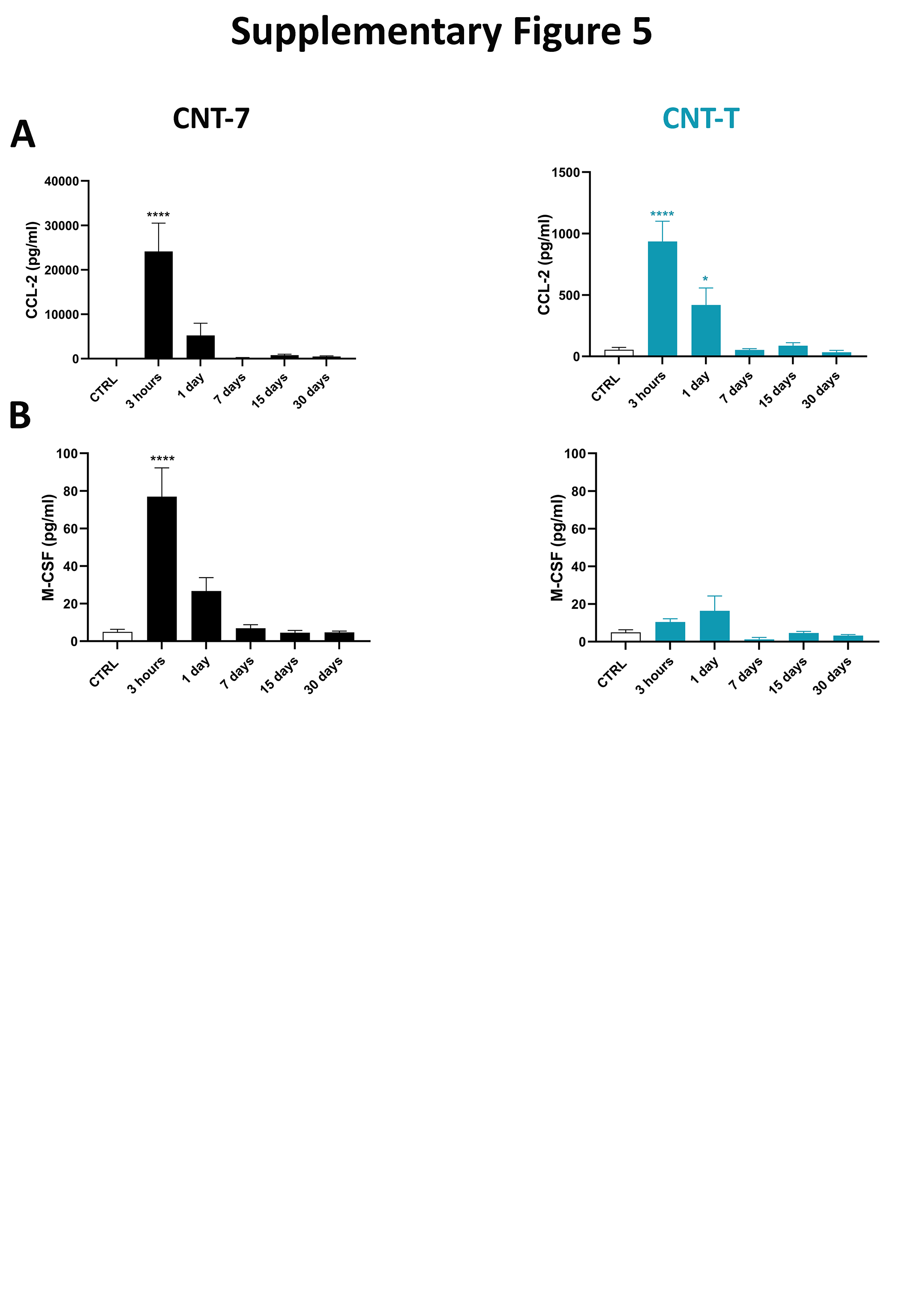

Supplement: Supplementary Figure 5 — Levels of monocytes-related cytokines in the peritoneal fluids after mesotheliomagenic CNT-7 and non-mesotheliomagenic CNT-T. (A) CCL-2 and (B) M-CSF released in untreated (CTRL) and CNT-7 or CNT-T-treated rat (2 mg, I.P.) peritoneal fluids at 3 hours and 1, 3, 7, 15 and 30 days after injection were tested using Bio-Plex multiplex assay. Each point represents the mean of 4 samples ± SD. The results were statistically analysed using ANOVA test followed by Dunnett’s test. (*) p = 0.0332, (****) p < 0.0001 indicates a statistically significant difference with controls. [file Image_5.tif]
